# Lung Perfusion Scintigraphy Early After COVID-19: A Single-Center Retrospective Study

**DOI:** 10.2967/jnmt.121.262440

**Published:** 2021-12

**Authors:** De Sajal, Ravina Mudalsha, Lukose Tinu, T. Ganga Ranganath, Sahu Dibakar

**Affiliations:** 1 Department of Pulmonary Medicine, All India Institute of Medical Sciences, Raipur, India; and; 2 Department of Nuclear Medicine, All India Institute of Medical Sciences, Raipur, India

**Keywords:** early–post-COVID-19 patients, D-dimer, lung perfusion scan, novel coronavirus disease 2019

## Abstract

The incidence of thromboembolic complications in coronavirus disease 2019 (COVID-19) infection is well recognized. The present study retrospectively evaluated the type and prevalence of lung perfusion defects in early–post-COVID-19 patients with hypoxia and was aimed to identify the risk factors for mismatched perfusion defects. **Methods:** We analyzed SPECT/CT images of 54 early–post-COVID-19 patients (44 men and 10 women). Logistic regression analysis was used to examine the risk. **Results:** The mean age of the study population was 55.4 y (range, 34–76 y). All received prophylactic anticoagulation from the day of hospitalization to the date of perfusion scanning. The median interval between COVID-19–positive reports and lung perfusion scanning was 22 d. Lung perfusion defects (of any type) were observed in most (87%). Twenty-three subjects (42.6%) had mismatched perfusion defects. Mismatched perfusion defects were segmental in 14 subjects (25.9%) and subsegmental in 11 (20.4%). Higher age was a risk factor for mismatched perfusion defects (odds ratio, 1.06; 95% CI, 0.99–1.13; *P* = 0.06). Subjects with a serum D-dimer level of at least 2,500 ng/mL on the day before the scan were not at higher risk for having mismatched perfusion defects (odds ratio, 1.14; 95% CI, 0.34–3.9; *P* = 0.83). **Conclusion:** Despite prophylactic anticoagulation, mismatched perfusion defects suggestive of pulmonary thromboembolism were observed. Serum D-dimer level in patients early after COVID-19 is a poor predictor of mismatched perfusion defects. Confirmed evidence of pulmonary embolism by imaging studies should support the decision to extend anticoagulant prophylaxis in post-COVID-19 patients.

## Visual Abstract

**Figure:**
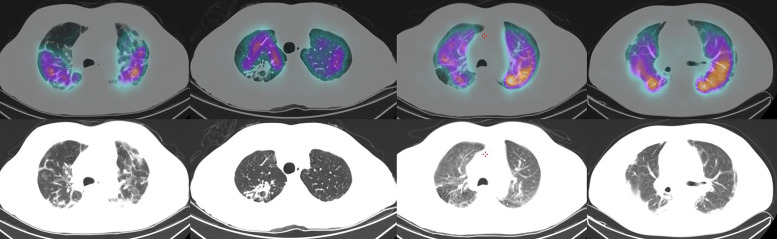

Severe acute respiratory syndrome coronavirus 2 (SARS-CoV-2), a novel member of the enveloped RNA β-coronavirus family, causes coronavirus disease 2019 (COVID-19). Most COVID-19 patients are either asymptomatic or mildly symptomatic.

The thrombogenic potential of SARS-CoV-2 infection is well recognized (*1*). The coagulation abnormalities, along with prolonged bed rest due to hospitalization, lead to high incidences of venous thromboembolism and thromboembolic complications, mostly pulmonary embolism (PE). Thromboprophylaxis with low-molecular-weight heparin is currently recommended for the treatment of hospitalized COVID-19 patients. Despite adequate anticoagulant therapy, both venous thromboembolism and PE, especially in severe and critically ill COVID-19 patients, have been reported (*1*). An observational study of over 500 COVID-19 patients admitted to 8 intensive care units in France reported 22.7% thrombotic complications, mostly PE (*2*). A metaanalysis of over 7,000 COVID-19 patients showed that the pooled in-hospital incidence of PE in the general ward and intensive care unit was 14.7% and 23.4%, respectively (*3*). Currently, there is no recommendation on whether prophylactic anticoagulation should be used for COVID-19 patients with a raised level of serum D-dimer at the time of discharge.

Multidetector CT pulmonary angiography (CTPA) is the gold standard for diagnosing PE. A ventilation–perfusion scan is an alternative to CTPA for diagnosing PE. However, there is a potential risk of infection transmission during ventilation–perfusion scanning (*4*). Lung perfusion scintigraphy by SPECT/CT is a safe alternative for diagnosing PE, especially for COVID-19 patients. The role of SPECT/CT in diagnosing PE in COVID-19 patients has been established (*4*). A perfusion scan of the COVID-19 patient is useful for evaluating residual-clot burden and small-vessel injuries (*5*). The presence of at least 1 wedge-shaped peripheral perfusion defect estimated as at least 50% involvement of a pulmonary segment without a corresponding CT image abnormality is indicative of PE in COVID-19 (*4*).

After recovery from active infection, a few COVID-19 patients, especially those who were severely or critically ill, continue to have hypoxia. The causes of persistent hypoxia in early–post-COVID-19 patients are not fully understood. Impaired lung perfusion is considered one of the underlying pathophysiologic mechanisms. The presence of perfusion defects in early–post-COVID-19 patients with persistent hypoxia has not, to our knowledge, been previously investigated. The present study retrospectively evaluated the type and prevalence of lung perfusion defects in early– post-COVID-19 patients with hypoxia and was aimed to identify the risk factors for mismatched perfusion defects.

## MATERIALS AND METHODS

### Study Design and Participants

We conducted a retrospective analysis of 54 early–post-COVID-19 patients admitted to the pulmonary medicine department of our institute between August 2020 and March 2021. The Institutional Ethics Committee approved this retrospective study, and the requirement to obtain informed consent was waived. All subjects had microbiologically confirmed SARS-CoV-2 infection (either by reverse-transcription polymerase chain reaction or rapid antigen testing). After 2 consecutively negative reverse-transcription polymerase chain reaction results for SARS-CoV-2, these patients were shifted from the COVID-19 wards to the pulmonary ward for ongoing treatment of hypoxia. The study subjects were stratified into 2 categories: moderate COVID-19 (i.e., those who received low-flow [e.g., nasal cannula] or high-flow [e.g., face mask, nonrebreathing mask, and high-flow nasal cannula] oxygen therapy) and severe COVID-19 (i.e., those who received invasive or noninvasive ventilator support during active COVID-19 infection).

### Data Collection

The demographic characteristics, comorbidities, clinical profile, laboratory reports, and treatment received were extracted from the medical records.

### Imaging Protocol

After intravenous injection of 148–222 MBq (4–6 mCi) of ^99m^Tc-macroaggregated albumin containing 4–6 × 10^5^ particles, SPECT imaging with low-dose CT was performed with the patient supine. Planar imaging was done in multiple projections (anterior and posterior; right and left lateral; right anterior and posterior oblique; and left anterior and posterior oblique). The images were reconstructed in the transaxial, coronal, and sagittal views and were reviewed for perfusion defects. The mismatched perfusion defects in this study were based on a mismatch between CT and scintigraphy images. The perfusion defects were further categorized as mismatched segmental perfusion defects (a wedge-shaped peripheral defect involving ≥50% of a pulmonary segment in all 3 orthogonal planes and without corresponding parenchymal abnormalities in the CT images), mismatched subsegmental perfusion defects (at least 1 wedge-shaped peripheral defect involving <50% of a pulmonary segment in all 3 orthogonal planes and without corresponding parenchymal abnormalities in the CT images), matched segmental perfusion defects (any perfusion defect involving ≥50% of a pulmonary segment in all 3 orthogonal planes with corresponding parenchymal abnormalities [e.g., consolidation, ground-glass opacities, or fibrosis] in the CT images), or matched subsegmental perfusion defects (any perfusion defect involving <50% of a pulmonary segment in all 3 orthogonal planes with corresponding parenchymal abnormalities [e.g., consolidation, ground-glass opacities, or fibrosis] in the CT images).

### Statistical Analysis

The study variables were expressed as mean ± SD, median with interquartile range, and proportion. The odds ratio with 95% CIs was calculated using logistic regression analysis to examine the association of risk factors with mismatched perfusion defects (segmental, subsegmental, or both). The risk factors assessed in this study were age, sex, serum D-dimer level on the day before the perfusion study, interval (in days) between lung scintigraphy and positive COVID-19 reports, and severity of disease (categoric variables). The statistical analysis was performed with SPSS, version 20.0 (IBM). A *P* value of less than 0.05 was taken as statistically significant.

## RESULTS

### Demographic Profile

The total study population was 54, and most were men (*n* = 44, 81.5%). The mean age was 55.4 y (median, 56 y; range, 34–76 y). A history of diabetes mellitus, hypertension, and coronary artery disease was present in 19 (35.2%), 24 (44.4%), and 3 (5.6%) subjects, respectively. Fourteen subjects had moderate COVID-19 infections (25.9%), and 40 had severe COVID-19 infections (74.1%). All patients received low-molecular-weight heparin prophylaxis from the day of hospitalization to the date of the perfusion scan. The serum D-dimer level on the day before perfusion scanning was available for 44 subjects. The median serum D-dimer level was 3,540 ng/mL (interquartile range, 1,457–6,549 ng/mL, range, 500–15,000 ng/mL). The median interval between COVID-19–positive reports and lung perfusion scanning was 22 d (interquartile range, 15–33 d; range, 8–107 d). The single person with the highest interval was taking an oral apixaban tablet.

### Perfusion Scintigraphy

Lung perfusion defects (of any type) were observed in 47 subjects (87%). Matched perfusion defects were the commonest and were observed in 39 subjects (72.2%). The type and prevalence of perfusion defects are presented in Table 1. Perfusion defects did not differ between moderate and severe COVID-19. Mismatched perfusion defects were observed in 23 subjects (42.6%). Most cases (61%) of mismatched perfusion were segmental. Six subjects had both matched and mismatched segmental perfusion defects. More cases of mismatched segmental perfusion defects were in the right lung than in the left (8 vs. 4, *P* < 0.01).

**TABLE 1:** Distribution of Various Types of Lung Perfusion Abnormalities

Type of abnormality	Moderate COVID-19 (*n* = 14)	Severe COVID-19 (*n* = 40)	Total (*n* = 54)
Perfusion defects (any)	14 (100)	33 (82.6)	47 (87)
Mismatched perfusion defects	7 (50)	16 (40)	23 (42.6)
Segmental	3 (21.4)	11 (27.5)	14 (25.9)
Subsegmental	4 (28.6)	7 (17.5)	11 (20.4)
Both segmental and subsegmental	0	2 (5)	2 (3.7)
Matched perfusion defects	11 (78.6)	28 (70)	39 (72.2)
Segmental	6 (42.9)	13 (32.5)	19 (35.2)
Subsegmental	7 (50)	23 (57.5)	30 (55.6)
Both segmental and subsegmental	2 (14.3)	8 (20)	10 (18.5)
Both matched and mismatched perfusion defects	4 (28.6)	11 (27.5)	15 (27.8)

Data are number followed by percentage in parentheses.

There was no significant difference in serum D-dimer level between subjects with only mismatched segmental perfusion defects and subjects with only matched segmental perfusion defects (median, 3,700 vs. 3,804 ng/mL; *P* > 0.05). Logistic regression analysis showed that the risk of mismatched perfusion defects increased as age increased (odds ratio, 1.06; 95% CI, 0.99–1.13; *P* = 0.06). A longer interval between positive COVID-19 results and the scintigraphy study did not create a lower risk of mismatched perfusion defects (odds ratio, 0.66; 95% CI, 0.22–1.96; *P* = 0.5). The risk for mismatched perfusion defects in men was not higher compared than that in women (odds ratio, 1.14; 95% CI, 0.28–4.62; *P* = 0.9). Patients with severe COVID-19 were not at higher risk of having mismatched perfusion defects than were patients with moderate COVID-19 (OR, 0.67; 95% CI, 0.19–2.27; *P* = 0.5). Patients with a serum D-dimer level of at least 2,500 ng/mL before the day of the scan were not at higher risk of having mismatched perfusion defects (odds ratio, 1.14; 95% CI, 0.34–3.9; *P* = 0.83).

## DISCUSSION

The present study showed mismatched perfusion defects suggestive of PE in early–post-COVID-19 patients, despite receiving anticoagulation prophylaxis from the first day of hospitalization. Lung perfusion defects in COVID-19 were matched or mismatched, and the distribution was segmental or subsegmental. A higher age carried a higher risk for having mismatched perfusion defects.

SARS-CoV-2 binds with the angiotensin-converting enzyme 2 receptor present on the cell surface. Binding of the virus on angiotensin-converting enzyme 2 receptors leads to upregulation of angiotensin II and activation of the renin-angiotensin-aldosterone system. Both this system and angiotensin II enhance platelet activity and activate the coagulation cascade, as well as increasing the expression of interleukin 6 and other inflammatory markers, which further amplifies the coagulation cascade (*6*). Increased levels of clotting factors, with disruption of the normal homeostasis of vascular endothelial cells in COVID-19, lead to microangiopathy and thrombus formation. Lung autopsies of COVID-19 patients have demonstrated endothelial injury, with intracellular virus in the pulmonary vasculature, microangiopathy, and widespread thrombosis with occlusion of alveolar capillaries (*7*). Deposition of fibrin and thrombin in the pulmonary microvasculature leads to impaired lung perfusion. The subsegmental mismatched perfusion defects in our study confirm the small-vessel injuries.

CTPA helps to visualize clots within the pulmonary vasculature, and most investigators have used CTPA for diagnosing PE in COVID-19. A Dutch study observed that most PE diagnosed by CTPA in COVID-19 patients was in segmental or more proximal arteries (*8*). Another study found that most PE diagnosed by CTPA was subsegmental (93%) (*9*). Therefore, PE in COVID-19 involves both segmental and subsegmental pulmonary arteries. CTPA can miss the thrombus in distal subsegmental small vessels and be unable to assess tissue perfusion. Thus, there is a risk of underestimation of PE in COVID-19 by CTPA (*5*). Idilman et al. identified 25.8% perfusion defects on the dual-energy CT scans of 31 patients with mild to moderate COVID-19 (*10*). Most PE (75%) in their study was without macroscopic thromboembolism on CTPA. Except for a few case reports and small case series, lung perfusion defects, especially in early–post-COVID-19 patients, have not been much investigated (*11*–*13*). The prevalence of segmental mismatched perfusion defects—that is, PE—in our study was similar to that in a previous study (*3*). The longer interval between COVID-19–positive reports and scanning did not reduce the risk for mismatched perfusion defects. Therefore, despite anticoagulant prophylaxis, PE of COVID-19 takes longer to resolve. Mismatched perfusion defects in our study were more on the right side, similar to the observation by Mueller-Peltzer et al. (*14*).

Serum D-dimer is considered a sensitive test to diagnose thrombotic states, including PE. A systematic review and metaanalysis showed that serum D-dimer in severe COVID-19 was significantly higher than non-severe forms and correlated with the disease severity (*15*). In COVID-19 patients, the rise in serum D-dimer is due to activation of the blood coagulation cascade secondary to a systemic inflammatory response or as a direct consequence of the virus itself. Serum D-dimer in COVID-19 patients correlates poorly with the venous thromboembolism score (*16*). Several investigators tried to find the D-dimer cutoff that identifies the risk of PE in active COVID-19 infection. Cui et al. found that a serum D-dimer level of 1,500 ng/mL has 85.0% sensitivity and 88.5% specificity for predicting venous thromboembolism (*17*). Léonard-Lorant et al. observed that a serum D-dimer threshold of 2,660 μg/L detected all cases of PE by CTPA in COVID-19 patients (*18*). We observed that subjects with a serum D-dimer level of at least 2,500 ng/mL were not at higher risk of having mismatched perfusion defects. Therefore, an elevated level of serum D-dimer in early–post-COVID-19 patients is not necessarily attributable to underlying PE.

This study had several limitations. It was a single-center retrospective study, and not all early–post-COVID-19 patients with hypoxia were investigated. Serial serum D-dimer levels were not available for all patients. The number of subjects was small, and no patients with mild COVID-19 were enrolled.

## CONCLUSION

The present study showed that SPECT/CT images suggestive of PE were independent of serum D-dimer level before the day of scanning. An elevated level of serum D-dimer in early–post-COVID-19 patients should not be a criterion for posthospitalization anticoagulant therapy unless imaging studies confirm the PE. Carefully designed prospective studies are necessary to identify the post-COVID-19 patients who will require extended thromboprophylaxis.

## DISCLOSURE

No potential conflict of interest relevant to this article was reported.
